# Supplementary material for: Pressure-tuning of α-RuCl3 towards a quantum spin liquid
Source: Nat Commun. 2024 Sep 17;15:8142. doi: 10.1038/s41467-024-52169-w (PMC11408671; doi:10.1038/s41467-024-52169-w)
Supplement: Supplementary file 1 — Supplementary Information [file 41467_2024_52169_MOESM1_ESM.pdf]

Supplementary Information

SUPPLEMENTARY NOTE 1: X-RAY DATA ANALYSIS AND STRUCTURE REFINEMENT

The data collected at ambient conditions for crystal 1 was processed using Bruker's Apex3 software (Ref. 1), the reflection intensities were integrated using SAINT (Ref. 2) and multi-scan absorption correction was applied using SADABS (Ref. 3). The subsequent structure solution and weighted fullmatrix least-squares refinement on F^2 were done with SHELXT-2014/5 (Ref. 4) and SHELXL-2018/3 (Ref. 5) as implemented in the WinGx 2018.3 program suite (Ref. 6). Key details of the data collection and the structural refinement are summarized in Supplementary Table 1. The atomic positions and isotropic displacement parameter based on the single-crystal x-ray diffraction data at ambient conditions are listed in Supplementary Table 3.

To determine the averaged structure at 1.26 GPa and ambient temperature of crystal 2, the data was integrated and corrected for Lorentz, polarization and background effects using the CrysAlisPro software suite (version 171.39.46) [7]. Reflections, which were saturated due to overexposure or an overlap with diamond peaks, were omitted from the integration process. Note that the separation of sharp and diffuse scattering requires no special treatment, as the Bragg peaks of family 1 and 2 are not affected by diffuse intensity. We collected 135 reflections, which were merged based on the crystal symmetry to 30 independent reflections with $R_{int} = 4.69\,\%$. The averaged structure was then solved in the space group $p\bar{3}m1$ using the SHELXT-2014/5 (Ref. 4) and SHELXL-2018/3 (Ref. 5) as implemented in the WinGx 2018.3 program suite (Ref. 6). The final refinement converged at R_1 (all data) = 2.65 % and wR_2 (all data) = 5.31 %. The parameters characterizing the data collection and the structural refinement are summarized in Supplementary Table 2.

In accordance with previous reports we observed a transition into the triclinic dimerized phase at $p = 1.5$ GPa as illustrated in Supplementary Figure 1.

Supplementary Table 1. Details on data collection and structure refinement of α-$RuCl_3$ as determined from single-crystal X-ray diffraction at ambient conditions.

Crystal data		Data collection		Refinement	
Pressure (GPa)	0	*Wavelength* (Å)	0.7107	$N_{parameters}$	22
Temperature (K)	295	$2\theta_{max}$ (°)	60.88	$R_1 > 4\sigma$ (%)	1.62
Space group	$C2/m$	T_{min}	0.6214	R_1 *all* (%)	1.62
a (Å)	5.9875(6)	T_{max}	0.7461	$wR_2 > 4\sigma$ (%)	3.86
b (Å)	10.3529(3)	$N_{measured}$	2006	wR_2 *all* (%)	3.86
c (Å)	6.0456(6)	$N_{observed}$ $[I>2\sigma(I)]$	555	$\Delta\rho_{min}$ (e·A^{-3})	-0.773
β (°)	108.777(9)	μ (mm^{-1})	6.397	$\Delta\rho_{max}$ (e·A^{-3})	0.764
Z	4	R_{int} (%)	2.52	*G.O.F*	1.093
ρ_{calc} (g·cm^{-3})	3.883			*weight w (a,b)*	0.0195
				Extinction	0.0007

Supplementary Table 2. Details on data collection and structure refinement of α-$RuCl_3$ as determined from single-crystal X-ray diffraction at 1.26 GPa and ambient temperature.

Crystal data		Data collection		Refinement	
Pressure (GPa)	1.26	*Wavelength* (Å)	0.4113	$N_{parameters}$	6
Temperature (K)	300	$2\theta_{max}$ (°)	37.5	$R_1 > 4\sigma$ (%)	2.50
Space group	$P\bar{3}m1$	T_{min}	0.68	R_1 *all* (%)	2.65
a (Å)	3.4080(4)	T_{max}	1.00	$wR_2 > 4\sigma$ (%)	5.08
b (Å)	3.4080(4)	$N_{measured}$	135	wR_2 *all* (%)	5.31
c (Å)	5.562(10)	$N_{observed}$ $[I>2\sigma(I)]$	30	$\Delta\rho_{min}$ (e·A^{-3})	-0.458
Z	1	μ (mm^{-1})	9.863	$\Delta\rho_{max}$ (e·A^{-3})	0.685
ρ_{calc} (g·cm^{-3})	-	R_{int} (%)	4.69	*G.O.F*	1.404
				weight w (a,b)	0.0191

Supplementary Table 3. Fractional atomic coordinates and equivalent isotropic displacement parameters (Å^2) of a α-$RuCl_3$ single crystal at ambient conditions.

Atom	Site	x	y	z	U_{eq}
Ru	4h	0	0.16651(2)	0.5	0.00964(9)
Cl_1	4i	0.22680(13)	0	0.73488(12)	0.01431(14)
Cl_2	8j	0.25058(10)	0.17411(4)	0.26761(9)	0.01407(12)

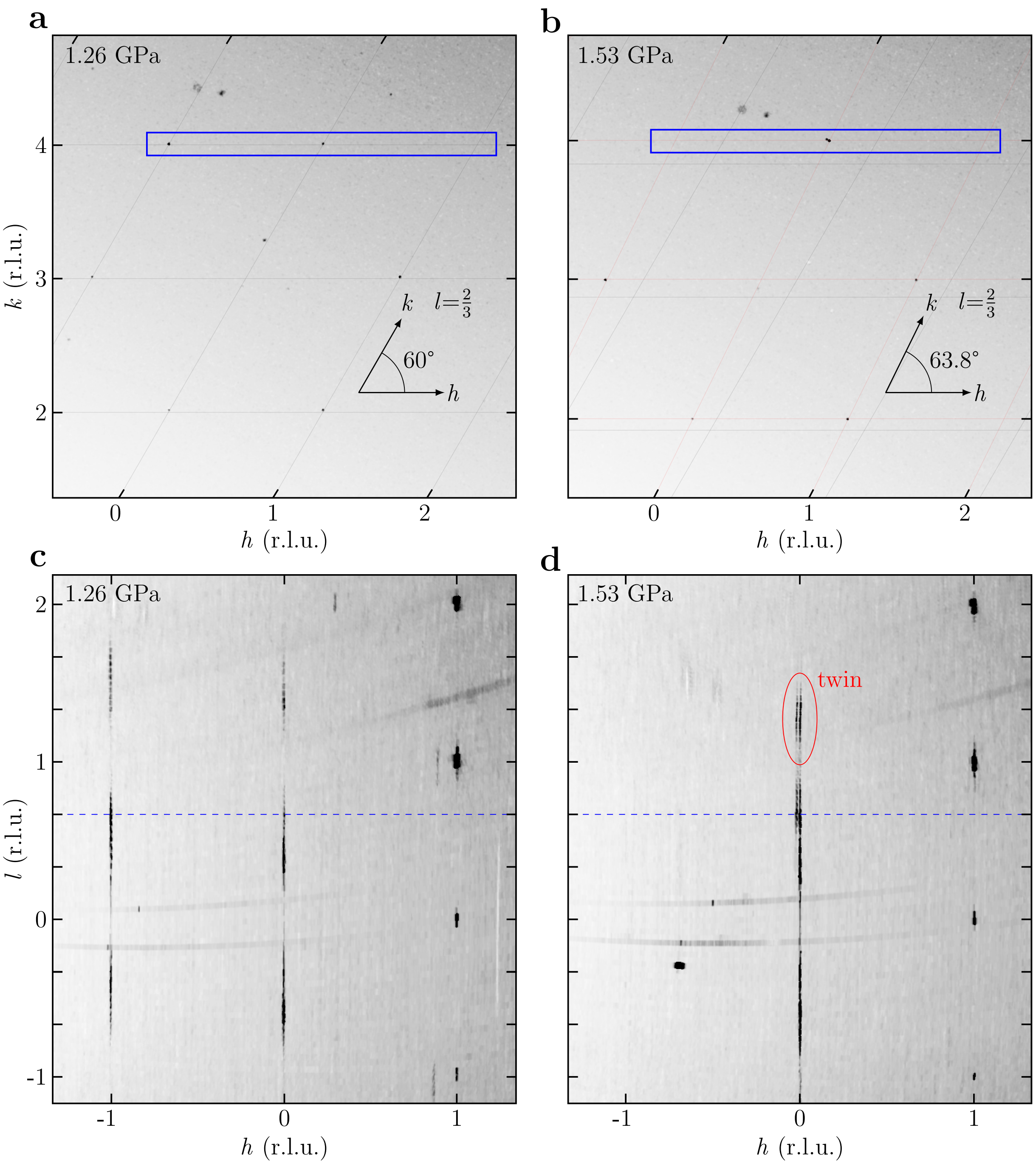

Supplementary Figure 1. Transition into the triclinic dimerized phase: (a and b): Reciprocal space maps for the hk-plane at $l = 2/3$. Gray solid lines in (a) and (b) indicate the hexagonal lattice of the high-symmetry phase at $p = 1.26$ GPa. Red lines in (b) indicate the triclinic lattice of the dimerized phase with the angle between h and k being 63.8°. (c and d): Reciprocal space maps for the hl-plane at $k = 2$ (this region is indicated by blue rectangles in (a) and (b)).

SUPPLEMENTARY NOTE 2: EXTRACTION OF THE DIFFUSE INTENSITIES

The diffuse intensity profiles were extracted from the diffraction data collected with a MAR555 flat panel detector at ID15B of the ESRF. The diffraction images were transformed into reciprocal space and $\left|F_0^2\right|$ maps were reconstructed by applying Lorentz and polarization factors using the CrysAlisPro software package [7]. For further data processing the Python packages numpy, matplotlib, and fabio were applied. The intensity profiles were estimated from the reconstructed layers for each pixel row along l by adding the pixel values for all pixels lying within the peak region $h, k \pm$ 0.02 and then subtracting a background intensity. The background intensity was as well determined line-by-line along l by calculating the average intensity in regions $\Delta k = 0.03$ immediately adjacent to the peak region.

SUPPLEMENTARY NOTE 3: MODELLING OF THE DIFFUSE SCATTERING

First, a single layer was built up by expanding the hexagonal unit cell to a 20x20x1 supercell. For the simulation 1000 of these layers were stacked along c_h. There are various possibilities to stack the individual layers, while preserving a hexagonal or cubic closed packing of the Cl-atoms. Altogether, there are 9 possibilities for the orientation of two adjacent layers that meet these requirements. The configuration of a single layer is specified by the position A1, A2, ..., C3 of the octahedral voids within the hexagonal cell, as illustrated in Supplementary Figure 2. In order to depict the stacking of the layers a stacking vector $\mathbf{T}_S$ is implemented, which connects the octahedral voids in successive layers. The stacking vector $\mathbf{T}_S$ with S=A1,A2,...C3 points from the octahrdal void A1 in the initial layer to an octahedral void S in the successive layer. In the event of a hexagonal closed packing of the Cl-atoms the arrangement options for two consecutive layers is reduced to three. We define these three kinds of stacking as *eclipsed* (e), *forward* (f) and *backward* (b), that correspond to the stacking vectors $\mathbf{T}_{A1}$ = [0,0,1], $\mathbf{T}_{A2}$ = [2/3,1/3,1] and $\mathbf{T}_{A3}$ = [1/3,2/3,1] respectively. However, our analysis showed that the measured XRD-data is very well reproduced by the exclusive use of f and b stacks. In order to probe the short-range order in the pressurized α-$RuCl_3$ samples, the sequence of f and b stacks in our simulation is generated by a first-order Markov process. To resemble the experimental found stacking disorder two independent transition probabilities p_{ff} and p_{bb} must be defined. Here, p_{ff} corresponds to the probability that a layer be f stacked on a preceding f stack, and accordingly p_{bb} is the probability of continuing in a *backward* stacking sequence. Therefore the probabilities for the presence of a stacking fault are p_{fb}=1-p_{ff} and p_{bf}=1-p_{bb}, respectively. The process is characterized in form of a right stochastic matrix $\mathbf{P}$ containing the transition probabilities.

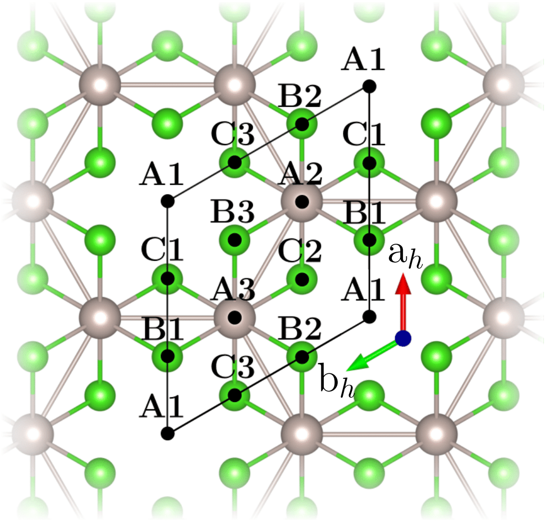

Supplementary Figure 2. Plane view of one Cl–Ru–Cl layer with in-plane basis vectors a_h and b_h, and illustrated Ru honeycomb net. The A1, A2, ..., C3 notation is used to specify the nine different possibilities of stacking two adjacent layers on top of each other according to the position of the octahedral voids within a single layer.

$$\mathbf{P} = \begin{pmatrix} p_{ff} & p_{fb} \\ p_{bf} & p_{bb} \end{pmatrix}$$

Using this approach DISCUS creates a list of layer positions. The scattering intensity of the layered model crystal is finally calculated as the product of the individual Fourier transform of the layer positions and the single layer. All in all we created 676 different disorder models by adjusting the transition probabilities p_{ff} and p_{bb} in steps of 0.04. For each model we calculated the intensity profiles along $12l$, 1-$3l$ and 1-$4l$ over the range -2 $\leq l \leq$ +2. Obviously the model crystal contain far fewer layers than do real crystals and consequently the number of stacking fault events is small. To reduce the statistical noise and thus produce a smooth intensity distribution along l the line profiles for each set of transition probabilities p_{ff} and p_{bb} were simulated 200 times and merged. To obtain quantitative agreement between simulated and measured XRD-data, the calculated diffuse profiles were adjusted by a scale factor to the observed intensity profiles.

The examination of the simulated intensity profiles revealed that the stacking probabilities and the x coordinate of the Cl-atom have significantly different influence on the distribution of the intensity along l, and can therefore be determined separately.The probabilities of finding a t or f stack determine the shape and position of the peak maxima. Even small variations in the probability values induce discernible effects on the simulated intensity profiles. We estimate the uncertainties of the optimized values $p_{ff} = 0.60$ and $p_{bb} = 0.72$ to be ± 0.02. In contrast, the relative intensities with a period $l = n + 1$ along the streaks are mainly dependent on the x coordinate of the Cl-atom, which is particularly evident for the intensity maxima located at $l = n + 1/3$ and $l = n + 2/3$. The simulation was carried out for several Cl-positions Δx displaced in 3 pm steps parallel to a_h from the average position $x_{Cl} = 1/3$ ($\Delta x =$ 0 pm) deduced from the sharp reflections. The results are shown in Figure 4 (a)-(c) by solid lines. Note that only the position of the Cl-atom is changed for the simulated profiles shown in Figure 4 (a)-(c), while the stacking fault probabilities remain unaltered.

SUPPLEMENTARY NOTE 4: DENSITY FUNCTIONAL THEORY

We thoroughly analyzed the effects of different DFT approximation levels on the symmetry-constrained structural relaxations. We compared the structures obtained from plain GGA, fully relativistic GGA+SOC and fully relativistic GGA with on-site Hubbard correction GGA+SOC+U. Additionally, we also studied the effect of empirical DFT-D2 van der Waals dispersion-corrections as implemented in the quantum-espresso package [8, 9]. We used $U = 1.5$ eV for the U parameter as in previous DFT studies on this material [10]. To keep the numerical effort reasonable we sampled the Brillouin zone on mesh a of $8 \times 8 \times 8$ k-points and used the Marzari-Vanderbilt-DeVita-Payne smearing with a smearing parameter of 0.02 Ry. In each calculation the symmetry constrained atomic positions within the unit cell have been relaxed until the atomic forces were smaller than 0.001 Ry/Bohr.

The structures obtained from these calculations are shown in the following Supplementary Tables 4 and 5. As can be clearly seen the different DFT approximation levels (GGA, GGA+SOC, GGA+SOC+U) yield very similar optimized structures with the larges differences occurring from plain GGA to GGA+SOC. In particular, the crucial Cl-Ru-Cl binding angle varies only within $\approx$ 0.3 as a function of the Hubbard U and the DFT-D2 dispersion corrections. Overall, the optimized DFT(+SOC+U) structures are very close to the experimentally found structure. We have also verified the result for the R3b high-symmetry phase with the full potential FPLO DFT-code [11, 12] by calculating the total Energy as a function of δx_{Cl}

	DFT		GGA+SO		GGA+SO+U (U=1.5 eV)	
	no vdW corr	DFT-D2	no vdW corr	DFT-D2	no vdW corr	DFT-D2
Ru						
x	0	0	0	0	0	0
y	0.16643	0.16651	0.166327	0.166458	0.166497	0.166499
z	1/2	1/2	1/2	1/2	1/2	1/2
Cl_1						
x	0.22099	0.22282	0.22618	0.2279	0.22686	0.22841
y	0	0	0	0	0	0
z	0.72955	0.73055	0.73482	0.73573	0.73515	0.73644
Cl_2						
x	0.24985	0.25020	0.25005	0.2504	0.2504	0.25032
y	0.17636	0.17558	0.17466	0.17385	0.17416	0.17367
z	0.27259	0.27134	0.26788	0.26667	0.26738	0.26594
Ru-Ru (Å)	3.44646	3.44774	3.44394	3.44664	3.44745	3.44749
	3.45765	3.45701	3.45891	3.45756	3.45716	3.45714
Ru-Cl-Ru (°)	94.9874	94.6555	93.6332	93.3582	93.5693	93.2217
	95.4547	95.0348	94.2565	93.8374	94.0206	93.6698

Supplementary Table 4. Optimized atomic positions for the monoclinic phase (C2/m) for different levels of DFT approximation. Atomic coordinates refer to the conventional monoclinic cell with lattice parameters $a_m = 5.9875$ Å, $b_m = 10.3529$ Å, $c_m = 6.0456$ Å and $\beta = 108.777$. The column highlighted in red corresponds to the GGA+SOC calculations that led to the structural parameters given in the original version of the manuscript.

	DFT		GGA+SO		GGA+SO+U (U=1.5 eV)	
	no vdW corr	DFT-D2	no vdW corr	DFT-D2	no vdW corr	DFT-D2
Ru						
x	2/3	2/3	2/3	2/3	2/3	2/3
y	1/3	1/3	1/3	1/3	1/3	1/3
z	0.49988	0.49991	0.50002	0.50002	0.49963	0.49975
Cl_1						
x	0.347148	0.345363	0.34326	0.341589	0.342993	3.41484
y	0.00024	0.00011	0.00024	0.00038	0.00037	0.00031
z	0.737576	0.738206	0.74149	0.742030	0.742261	0.742629
Ru-Ru (Å)	3.40799	3.40799	3.40797	3.40799	3.40799	3.40799
Ru-Cl-Ru (°)	93.8158	93.5042	92.7325	92.4561	92.5846	92.3444

Supplementary Table 5. Optimized atomic positions for the high symmetry phase (R3b) for different levels of DFT approximation. Atomic coordinates are mapped to the hexagonal unit cell of the layer space group P-3m1 ($a_h = b_h = 5.9028$ Å, $c_h = 5.9028$ Åand $\alpha = \beta = 90, \quad \gamma = 120$). The column highlighted in red corresponds to the GGA+SOC calculations that led to the structural parameters given in the original version of the manuscript.

[1] Bruker AXS Inc., “Apex3 v2018.1-0,” (2017).
[2] Bruker AXS Inc., “Saint(v8.30a),” (2017).
[3] L. Krause, R. Herbst-Irmer, G. M. Sheldrick, and D. Stalke, Journal of Applied Crystallography **48**, 3 (2015).
[4] G. M. Sheldrick, Acta Crystallographica Section A Foundations and Advances **71**, 3 (2015).
[5] G. M. Sheldrick, Acta Crystallographica Section C Structural Chemistry **71**, 3 (2015).
[6] L. J. Farrugia, Journal of Applied Crystallography **45**, 849 (2012).
[7] Rigaku Oxford Diffraction, “CrysAlisPro Software system version 1.171.39.46,” (2018).
[8] V. Barone, M. Casarin, D. Forrer, M. Pavone, M. Sambi, and A. Vittadini, Journal of Computational Chemistry **30**, 934 (2009), https://onlinelibrary.wiley.com/doi/pdf/10.1002/jcc.21112.
[9] S. Grimme, Journal of Computational Chemistry **27**, 1787 (2006), https://onlinelibrary.wiley.com/doi/pdf/10.1002/jcc.20495.
[10] S. Biswas, Y. Li, S. M. Winter, J. Knolle, and R. Valentí, Phys. Rev. Lett. **123**, 237201 (2019).
[11] K. Koepernik and H. Eschrig, Phys. Rev. B **59**, 1743 (1999).
[12] H. Eschrig, M. Richter, and I. Opahle, *Relativistic Solid State Calculations, in: Relativistic Electronic Structure Theory, Part 2. Applications*, edited by P. Schwerdtfeger, Vol. 13 (Elsevier, 2004) pp. 723–776.
